# A Spatial-Temporal Graph Convolutional Recurrent Network for Transportation Flow Estimation

**DOI:** 10.3390/s23177534

**Published:** 2023-08-30

**Authors:** Ifigenia Drosouli, Athanasios Voulodimos, Paris Mastorocostas, Georgios Miaoulis, Djamchid Ghazanfarpour

**Affiliations:** 1 Department of Informatics and Computer Engineering, University of West Attica, 12243 Egaleo, Greece; idrosouli@uniwa.gr (I.D.);; 2 Department of Informatics, University of Limoges, 87032 Limoges, France; 3 School of Electrical and Computer Engineering, National Technical University of Athens, 15773 Athens, Greece

**Keywords:** transportation flow estimation, graph convolutional networks, spatial-temporal dependencies, LSTΜ, deep learning, metro dataset, bike-sharing system dataset

## Abstract

Accurate estimation of transportation flow is a challenging task in Intelligent Transportation Systems (ITS). Transporting data with dynamic spatial-temporal dependencies elevates transportation flow forecasting to a significant issue for operational planning, managing passenger flow, and arranging for individual travel in a smart city. The task is challenging due to the composite spatial dependency on transportation networks and the non-linear temporal dynamics with mobility conditions changing over time. To address these challenges, we propose a Spatial-Temporal Graph Convolutional Recurrent Network (ST-GCRN) that learns from both the spatial stations network data and time series of historical mobility changes in order to estimate transportation flow at a future time. The model is based on Graph Convolutional Networks (GCN) and Long Short-Term Memory (LSTM) in order to further improve the accuracy of transportation flow estimation. Extensive experiments on two real-world datasets of transportation flow, New York bike-sharing system and Hangzhou metro system, prove the effectiveness of the proposed model. Compared to the current state-of-the-art baselines, it decreases the estimation error by 98% in the metro system and 63% in the bike-sharing system.

## 1. Introduction

With the rapid development of modern cities, population growth, and technological upgrading and development (Artificial Intelligence, Internet of Things, and Internet of Vehicles [1]), there has been an urgent need for more sophisticated and efficient transport systems both in terms of their structure and their organization and operation.

Traffic flow estimation is one of the critical issues of modern transportation management and Intelligent Transportation Systems (ITSs) structure. It contributes to the planning of travel routes, the evaluation of travel demand, the effective and efficient operation of public transport and, consequently, to the efficient operation of the whole city and the alleviation of transportation-related problems.

Cities transport sector is expanding and altering to become more accessible, to be able to serve larger numbers of passengers without compromising quality of traveling, and to become more ecologically conscious and sustainable. Modern public transport systems include road vehicles, underground railways, as well as various other new modes of transport that have appeared in recent years, including bike-sharing systems, ride-hailing services, and e-scooter.

The underground transit seems to be the leading force in public transportation. Worldwide investments in underground rail transportation infrastructure are growing fast every year [2]. Most cities without metro are planning to construct one, and the cities that have already developed subways are building new tracks and expanding their subway network due to everyday traffic pressure. The speeds of movement, the accuracy of the transition times, as well as an extensive network of stations are some of the reasons why it excels over other public transportation media.

In addition to underground railway transit, shared bicycles seem to be one of the best alternatives to address the climate emergency, traffic road congestion, and overcrowding. A bike-sharing system consists of bicycles which are available to the citizens to rent them for short or long distances. Thus, several stations are strategically distributed throughout the city, in order to allow people to rent and return the bikes according to their traveling needs. Bike-sharing systems have already been implemented in numerous places throughout the world.

Therefore, the transport network has become more complex and difficult to manage. Due to the irregular structure of the transportation network and the fluctuating temporal features of traffic flow, the transportation flow forecasting problem seems to be more challenging than other traditional time series forecasting problems which have a simpler structure and complexity. The traffic state in a specific location has both spatial and temporal dependencies, and thus it is important to take both of them into account in order to make an accurate estimation.

In recent years, there has been a growing interest in employing a range of methodologies to address prediction problems within the transportation domain. These approaches encompass a diverse set of techniques. Initially, statistical models such as ARIMA [3] and its extensions, such as ARIMAX [4] and SARIMA [5,6], were employed. However, these models assume only linear relationships between variables, which led to their replacement by traditional machine learning methods [7,8] that could address this limitation. Subsequently, deep learning methods [9,10,11] gained prominence as they were able to capture complex non-linear spatial-temporal dependencies, which are a significant characteristic in transportation data. Moreover, it is worth noting that recent advancements in transportation research have introduced innovative applications of deep learning techniques. For instance, an innovative platform for data acquisition (vehicle trajectory extraction) and analytics (reconstruction and evaluation) has been created specifically for Automated Driving System (ADS). This platform takes advantage of the power of deep learning algorithms in order to effectively improve transportation safety [12]. Additionally, the introduction of HYDRO-3D, a hybrid object detection and tracking system utilizing 3D LiDAR, has successfully used deep learning models such as Transformers and U-net to improve cooperative perception in transportation scenarios [13]. Recently, Graph Neural Networks (GNNs) have been used in order to make full use of the topological features of the transportation graph and capture the transportation network’s spatial dependencies.

In this work, we present a deep learning framework for transportation flow estimation using historical data elicited from users’ mobility services. This estimation model learns from both the spatial stations network data and time series of historical mobility changes in order to forecast transportation flow at a future time. The model is based on Graph Convolutional Networks (GCN) and Long Short-Term Memory (LSTM) networks in order to further improve the accuracy of transportation flow estimation. Extensive experiments on two real-world datasets of transportation flow, Hangzhou metro system and New York bike-sharing system, show the effectuality of the model.

The remainder of the paper is structured as follows: Section 2 provides a concise summary of related studies, while Section 3 introduces and describes the Spatial-Temporal Graph Convolutional Recurrent Network (ST-GCRN) model. Our proposed model is extensively evaluated in Section 4, where it is compared to several other baseline methods. The paper concludes in Section 5 with a summary of our findings.

## 2. Related Work

Transportation flow estimation is a significant research problem in the intelligent transportation field. There have been several methods to predict transportation flow in systems, such as metro, bus, bike, train, etc.

Statistical methods: Traditional methods are mainly based on mathematical statistical models which use the techniques of time series analysis in statistics. Autoregressive Integrated Moving Average (ARIMA) [3] and its variants [4,5] are the most effective approaches designed to predict future values in short-term traffic data based on historical observations. In [6], an ARIMA model has been used to predict the passenger flow of Guangzhou metro based on the historical passenger flow data collected by the ticketing automatic system of urban rail transit. In [14], the rule of passenger flow in and out of Beijing subway station is analyzed in accordance with time changes, and the SARIMA model is used for modeling.

Although these methods allow for transportation pattern identification, they assume linear relationships between variables and that past pattern will repeat in the future. Thus, complex multivariate data of modern transportation systems are not processed effectively and non-linear relationships between variables are difficult to capture.

In order to address these issues, researchers eventually switched from statistical approaches to machine learning and deep learning models due to the intricate relationships of transportation flow data.

Traditional machine learning methods: Various machine learning methods are used in order to analyze heterogeneous data from various sources [15,16,17]. Numerous studies have been conducted to forecast the passenger flow in various transportation media. In [8], a hybrid model is used that combines K-means algorithm for clustering the original sample set and Support Vector Machine (SVM) to forecast the public bicycle traffic flow. A method to extract passenger flow of different routes on bus stations is implemented by using an XGBoost model in [18], whereas in [19], a Multi-Feature Gradient Boosting Decision Tree (GBDT) model is proposed in order to accurately predict short-term bus passenger flow.

Deep learning methods: Deep learning methods have demonstrated significantly higher effectiveness in predicting passenger flow in transportation media when compared to ML or statistical techniques [20,21]. These methods are able to represent complex non-linear spatial-temporal dependencies which are a major characteristic in transportation data.

In [22], the article proposes a hybrid prediction method called ST-LSTM for accurate and real-time network traffic prediction. It combines the Savitzky–Golay filter, Temporal Convolutional Network (TCN), and Long Short-Term Memory (LSTM) to capture short-term local features and long-term dependence in network traffic data. This approach addresses the challenges of capturing the non-linear characteristics of large-scale network sequences and can be readily implemented in various industrial areas, such as smart cities, edge computing, cloud computing, and data centers. Similarly, the research in [23] introduces a hybrid prediction model that combines an encoder–decoder neural network with Long Short-Term Memory (LSTM) and a Savitzky–Golay filter. The model is designed to forecast future time series of water quality. To enhance the accuracy of the predictions, a Savitzky–Golay filter is applied to remove noise from the time series data. Additionally, an encoder–decoder model based on LSTM is utilized to capture relevant features for the prediction task.

In the process of passenger flow prediction, Recurrent Neural Networks (RNN) can effectively solve the problem of randomness and non-linearity which cannot be solved by the existing linear models. In [24], the combination of RNNs and wavelet transform is employed to predict the passenger flow and the results show that the method can effectively improve the prediction accuracy. Regular RNNs drawback is the vanishing gradient issue which means that part of the data from previous layers is lost. Long Short-Term Memory (LSTM) was used frequently to anticipate passenger movement as these models seem to address the vanishing gradient problem. A deep irregular convolutional LSTM network model called DST-ICRL for urban traffic passenger flow prediction was used in [9], whereas in [10], an end-to-end deep learning architecture based on the LSTM, termed as Deep Passenger Flow (DeepPF), managed to forecast the metro inbound and outbound passenger flow. LSTM models primarily take into account the temporal aspects of transportation flow. However, they do not take into account the limitations of network topology on transportation data changes.

Convolutional Neural Networks (CNNs) have been proven to extract spatial dependencies of transportation and spatial correlations. For predicting an urban rail transit passenger flow time series and spatial-temporal series, two deep learning neural networks were utilized in [11], a Long Short-Term Memory Neural Network (LSTM NN) for time series prediction and a Convolutional Neural Network (CNN) for spatial-temporal series prediction.

Although results of deep learning models seem promising to represent the non-linearity of transportation flow prediction, there are still some limitations considering the criticality of missing data [25] as well as the requirement of large amounts of historical data for training the model [26], which may result in over-fitting of the model due to fluctuations in a small time interval transportation flow. Both limitations are crucial for transportation flow forecasting, as data in this field must be accurate in order to lead to an effective performance.

Graph Neural Networks: Due to the recent continuous development of graph neural networks [27,28], researchers have taken into consideration the graph structure of transportation networks and have started to use GNN-based methods for transportation flow prediction tasks.

With graph neural networks which are special types of neural networks capable of processing graph structured data in non-Euclidean space [29], spatial and temporal dependencies can be learned, making more accurate transportation flow predictions.

Graphs have been utilized by numerous studies to capture spatial-temporal dependencies. In [30], a new traffic prediction model called STGSA is introduced, which focuses on capturing both localized and long-term spatial-temporal dependencies. This model has the advantage of extracting hidden spatial-temporal features in a single step, eliminating the need for additional modules. Furthermore, it incorporates a heuristic spatial adjacency matrix optimization algorithm, enhancing its ability to capture trends in relevant nodes and incorporating valuable information. In addition, ST-Trader in [31] is a framework which takes into account the inter-connection of firms in order to predict stock prices. A Variational Autoencoder (VAE) was employed to reduce the dimensionality of the stock fundamental information and a clustering technique was applied to group stocks into a graph structure. A hybrid model called GCN-LSTM is proposed, which combines a Graph Convolutional Network (GCN) and a Long Short-Term Memory Network (LSTM). This model utilizes an adjacency graph matrix, obtained from the VAE, to forecast stock market trends in a graph-structured manner.

As in this study, two real-world transportation flow datasets have been utilized, one from Hangzhou metro railway system and one from NY bike-sharing system, and previous works are focused on these two areas of data.

Specific to bike-sharing system flow, the problem of accurate bike demand estimation in bike-sharing systems was investigated in [32] for effective station rebalancing by building a Spatial-Temporal Graph Neural Network (ST-GNN) model to predict the city wide bike demands. Similarly in [33,34], a model is built in order to predict flow at station level by viewing the bike-sharing system from the graph perspective and taking into account external influential factors, such as events [33], weather [34], etc. In [33], the attention-based mechanism is introduced to further improve the performance of a model which predicts the number of available bikes in bike-sharing systems in cities.

Various remarkable works have been performed in metro railway systems, as well. In [35], a dynamic spatial-temporal hypergraph neural network is proposed to forecast passenger flow. Furthermore, hypergraph convolution and spatial-temporal blocks are proposed to extract spatial and temporal features to achieve node level prediction. The model in [36] integrates the Relational Graph Convolutional Network (R-GCN), split-attention mechanism, and Long Short-Term Memory (LSTM) to explore the spatial-temporal correlations and dependence between passenger inflow and outflow.

Based on a priori knowledge, [37] creates multi-view graphs to express the static feature similarity of each station in the metro network, which are then inputted into the multi-graph neural network in order to realize the complex spatial dependence of each station’s passenger flow by extracting and aggregating features.

In summary, there have been a number of approaches in this field for predicting traffic flow based on conventional statistical machine learning and deep learning models. However, a number of major challenges still exist:

1. When the station network does not have a fixed structure but is dynamic, the relationship between the stations also changes. The spatial dependencies depend not only on the physical connections of stations, but also on the dynamics of the system, i.e., the mobility of flow makers (passengers or bikes), which depends on various external factors (weather, peak hours, personal choices, etc.). Therefore, it is a challenge to capture and take into consideration the dynamic spatial dependency relations between stations in order to make an accurate estimation of transportation flow.
2. The estimation of transportation flow in transport is highly dependent on whether the station network is structured or unstructured. Predicting flow is simpler in a structured network, where travel patterns are mostly predetermined, compared to an unstructured network where station usage is more random and fluctuates significantly over time. Thus, it is crucial to examine and contrast the estimation of transportation flow in both types of systems.
3. To enhance the accuracy of estimations and ensure their practical applicability, it is crucial to forecast not only in the short term, but also in the long term. As the duration of the estimation period increases, the impact of uncertain factors results in a decrease in the accuracy of predictions. Additionally, the dynamic variability of transportation flow further elevates the uncertainty of estimations. Generally, long-range predictions are more demanding than short-range ones, but their practical significance is greater. Thus, it is a challenge to attain a long-term estimation of transportation flow.
4. In a smart modern city, there are various modes of transportation, each with its unique traits and specific features. Therefore, it is essential that traffic prediction methods are comprehensive and not restricted to a single type of transportation mode.

To address the aforementioned challenges, we suggest a Spatial-Temporal Graph Convolutional Recurrent Network (ST-GCRN) for estimation of transportation flow. This technique can detect the dynamic spatial correlation between these stations and can perform long-term forecasting.

The main contributions of this paper are summarized as follows:

- Dynamic Relation Graph: Our research introduces a novel approach by proposing a dynamic relation graph to capture the changing spatial connections between stations. In contrast to traditional methods that rely solely on the physical topological layout, our approach incorporates the transportation flow within the system to determine the connections. This dynamic graph provides a more accurate representation of the actual relationships between stations, enhancing the estimation of transportation flow.
- Assessment of Different System Structures: We evaluate the effectiveness of our approach using two types of transportation flow datasets with different network structures. Specifically, we examine and contrast the estimation of transportation flow in a subway system, characterized by a more orderly arrangement of stations, and a bike rental system with a relatively less structured network. By considering these diverse systems, we demonstrate the versatility and applicability of our approach in different transportation contexts.
- Multi-Time Horizon Estimation: To enhance the accuracy and practical applicability of our estimations, we extend the analysis beyond short-term predictions. In addition to short-term estimations (15 and 30 min), we estimate future transportation flow in the long term (60 min) and various future time horizons (one, two, and three time steps ahead from three previous time steps). This comprehensive approach allows for very long-term estimations, providing valuable insights for transportation planning and management.
- Extensive Experimental Evaluation: We conduct extensive experiments on real-world datasets from two different transportation modes: the Hangzhou metro railway system and the NY bike-sharing system. Each dataset exhibits unique characteristics (as described in Section 4.1), and our approach, known as ST-GCRN, outperforms current state-of-the-art baselines in both systems. Specifically, our approach reduces the estimation error by 98% in the metro system and 63% in the bike-sharing system. These results demonstrate the superiority and practical effectiveness of our proposed method.

## 3. The ST-GCRN Model for Transportation Flow Estimation

Transportation flow domain space tackles data governed by complex relationships between stations which are spatially dependent and by dynamic temporal changes in each station. Leveraging spatial-temporal Graph Convolutional Networks (GCN) proves to be an optimal approach in this field, as it involves representing stations and connections between them as graphs, as well as forecasting transportation flow at each node station with a temporal prediction algorithm.

As indicated in [38], the spatial-temporal GCN is a type of graph neural network that has seen significant advancements recently due to its appealing efficiency, flexibility, and versatility.

To simultaneously capture the spatial and temporal dependencies from transportation flow data, we propose a Spatial-Temporal Graph Convolutional Recurrent Network (ST-GCRN) based on a Graph Convolutional Network (for learning spatial features) and a Long Short-Term Memory neural network (LSTM) (for predicting temporal components).

**Figure 1:**
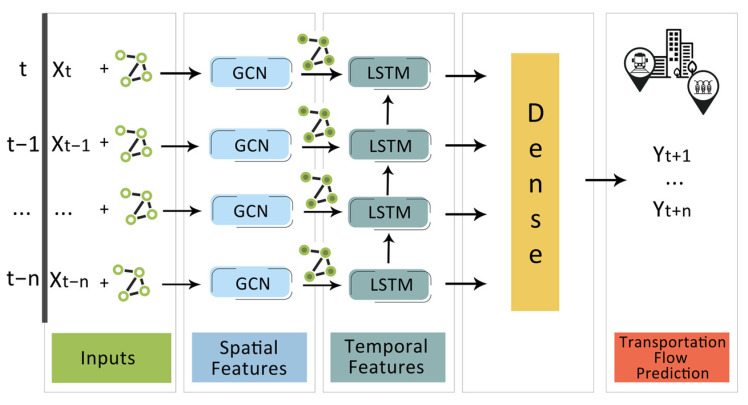
ST-GCRN model architecture. A graph convolution layer is applied to the inputs, followed by passing the results through an LSTM layer and a dense layer.

In this section, we describe how to utilize ST-GCRN model to implement transportation flow estimation. Specifically, ST-GCRN model consists of two parts: the GCN network and the LSTM layer. As depicted in Figure 1, the historical n time series data are used as input and the GCN is used to capture the topological structure of station network in order to acquire the spatial feature. In order to capture the temporal feature, the resulting time series with spatial features are then inputted into the LSTM model.

### 3.1. Definition of Transportation Flow Forecasting Problem

The transportation network in this work is presented as an undirected graph *G =* (*V, E, A*), where *V* represents the set of nodes (stations), *E* stands for the set of edges (physical or dynamic connections between stations), and *A* describes the adjacency matrix between stations. The adjacency matrix element *A_ij_* represents the connection relationship between two nodes, *v_i_* and *v_j_*. The element *A_ij_* in *A* equals 1 if node *i* and *j* are connected or 0 if otherwise. Therefore, adjacency matrix *A* indicates the neighbors of a node. Each node (station) has a number of features.

The purpose of transportation flow forecasting is to make predictions about future transportation flow based on past transportation flow for a specific period of time. The goal of this paper’s work is to construct a function f that takes historical transportation flow data *X_t_* as well as the graph *G*, as inputs and estimates the flow of all nodes at the next time, *X*′*_t + n_*:

*X*′*_t + n_* = *f* (*X_t_*, *G*) (1)

### 3.2. GCN Model

Graph Convolutional Networks (GCNs) are a type of neural network that operate on graph-structured data. The model equations for GCNs typically involve three main components: message passing, aggregation, and update operations.

Message Passing: In message passing, information is propagated through the graph by passing messages from neighboring nodes to update the node representations. The message passing equation can be defined as follows:

(2) $$\;h_{i}^{l}=\sigma \left(\sum_{j\in N_{i}}^{\;}\frac{1}{c_{ij}}W^{l}h_{j}^{l-1}\right).$$

where:

- $h_{i}^{l}$ represents the hidden representation of node *i* at layer *l*;
- *σ* denotes the activation function;
- $N_{i}$ represents the set of neighboring nodes of node *i*;
- $c_{ij}$ represents the normalization constant for the edge connecting nodes *i* and *j*;
- $W^{l}$ represents the learnable weight matrix for layer *l*.

Aggregation: After the message passing step, an aggregation operation is performed to combine the updated node representations into a single representation for each node. The aggregation equation can be defined as follows:

(3) $$a_{i}^{l}=\sigma \left(\sum_{j\in N_{i}}^{\;}h_{j}^{l}\right).$$

where $a_{i}^{l}$ represents the aggregated representation of node *i* at layer *l*.

These equations can be applied iteratively over multiple layers to capture increasingly complex patterns and dependencies in the graph data. The final node representations can then be used for various downstream tasks, such as node classification, link prediction, or graph-level predictions.

Update: All pooled messages are passed through an update function, usually a learned neural network.

### 3.3. LSTM Model

Long Short-Term Memory (LSTM) [39] is a type of Recurrent Neural Network (RNN) architecture that is designed to overcome the vanishing gradient problem and capture long-term dependencies in sequential data. It achieves this by using a memory cell and three main gates: the input gate, forget gate, and output gate. The equations for the LSTM model are as follows:

Input Gate *i_t_*:

(4) $$i_{t}=\sigma \left(W_{i}\;\left[h_{t-1},\;x_{t}\right]+b_{i}\right)$$

Forget Gate *f_t_*:

(5) $$f_{t}=\sigma \left(W_{f}\;\left[h_{t-1},\;x_{t}\right]+b_{f}\right)$$

Output Gate *o_t_*:

(6) $$o_{t}=\sigma \left(W_{o}\;\left[h_{t-1},\;x_{t}\right]+b_{o}\right)$$

Candidate Cell State ${\tilde{c}}_{t}$:

(7) $${\tilde{c}}_{t}=tanh\left(W_{c}\;\left[h_{t-1},\;x_{t}\right]+b_{c}\right)$$

Cell State *c_t_*:

(8) $$c_{t}=f_{t}\times c_{t-1}+i_{t\;}\times {\tilde{c}}_{t}$$

Hidden State *h_t_*:

(9) $$h_{t}=o_{t}\times tanh\left(c_{t}\right)$$

In the above equations:

- $i_{t}$ represents the input gate activation at time step *t*;
- $f_{t}$ represents the forget gate activation at time step *t*;
- $o_{t}$ represents the output gate activation at time step *t*;
- ${\tilde{c}}_{t}$ represents the candidate cell state at time step *t*;
- $c_{t}$ represents the cell state at time step *t*;
- $h_{t}$ represents the hidden state at time step *t*;
- $x_{t}$ represents the input at time step *t*;
- $W_{i},\;W_{f},\;W_{o},\;W_{c}\;$ are weight matrices, and $b_{i},\;b_{f},\;b_{o},\;b_{c}\;$ are bias vectors.

### 3.4. ST-GCRN Model

In our model, we use previous time steps as input features in order to predict the next one, two, or three time steps as the output. The spatial-temporal aspect results from the historical values of each feature for a specific station as well as the feature values of the stations which are connected to that specific station. The stations are connected physically (Hangzhou metro) or dynamically according to the stations usage (NYCBS).

As mentioned in Section 3.2, we use a graph *G =* (*V, E*) to describe the topological structure of the stations network. As described in Table 1, Hangzhou metro station network has 80 nodes and 6320 edges and NYCBS network has 50 nodes and 2450 edges, respectively.

In our work, we implement a neural network architecture which can process time series data over a graph. We first apply a graph convolution layer to the inputs and then we pass the results through a LSTM layer and a dense layer, as shown in Figure 1.

In our proposed framework, historical values of features on a number of previous time steps (*t − n, …, t − 1, t*) are used as inputs, in order to predict the transportation flow on a number of the next time steps (*t + 1, …, t + n*). Each node in the graph starts with an initial state and that state is updated through GCN layer, by receiving “messages” from the other nodes that are connected, as well. All the attribute vectors of any node in the graph are transformed by the application of an aggregation function. In our framework, the aggregation function is the mean value. Then, temporal features feed LSTM layer. Finally, the predicted values are produced from the dense layer.

## 4. Experimental Settings

In this section, the experimental evaluation will be presented. We will first describe the data used in experiments, the preliminary analysis on data, and the data preparation. Then, we make a brief reference to several popular models (including the current state-of-the-art) that will be used as baseline models to be compared to our proposed model framework, the metrics used for evaluation, and finally the hyper-parameters used in our model.

### 4.1. Data Description

Two real-world datasets, Hangzhou metro and bike NYCBS, are used to evaluate the performance of our method.

Hangzhou Metro System [40]: Hangzhou metro dataset was published by the Tianchi BigData Competition. It is a passenger flow (mobility) dataset which includes 25 days of subway card data files from 1 January 2019 to 24 January 2019, a total of about 70 million records from 81 subway stations on three lines. The number of samples, i.e., the outflow/inflow of passengers at a station at the various time intervals, is 2293.

NY City Bike System (NYCBS) [41]: The data are obtained from the NYC bike system from January 2021 to December 2022. Citi Bike is NYC’s official bike-sharing program, designed to give citizens an alternative to walking, taxis, buses, and subways. From the total number of stations, we use the 50 most frequently used. Moreover, there is a total of 14,848 samples, representing the number of bikes leaving or arriving at a station at each given moment.

The details of these two datasets are summarized in Table 2.

Each system in real world has its unique traits and specific features, which are briefly described in Table 3. The variations in the structure of each transport system, their adaptability to change, the level of predictability in travel patterns, and the time of usage and switching between modes of transportation result in distinct behaviors of each system. As a consequence, each system demands a unique approach.

### 4.2. Preliminary Data Analysis

The features of each dataset that are used in our approach are depicted in Table 4 and Table 5.

**Figure 2:**
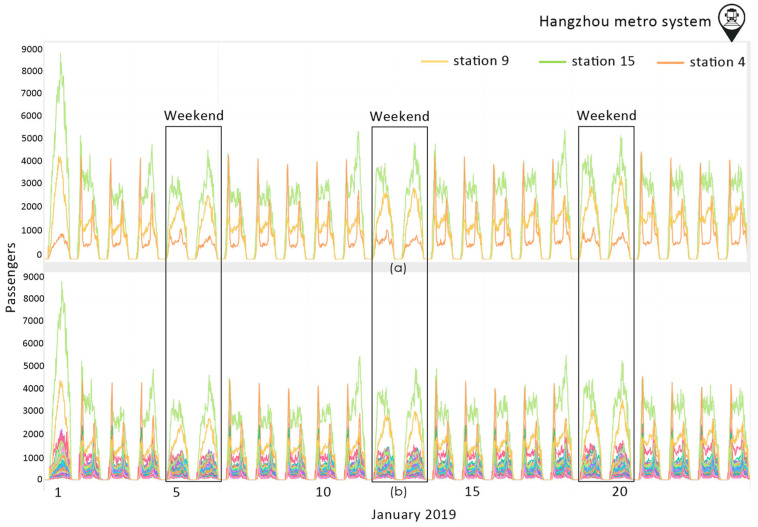
The transportation flow through time in Hangzhou metro system dataset (**a**) for the three most frequently used stations; (**b**) for all stations. The passengers flow follows a certain pattern on weekdays. During weekends, there is a shift in the pattern, with the peak hours occurring in the afternoon. Different color lines refer to different stations.

The transportation flow through time in Hangzhou metro system dataset is presented in Figure 2. We can see that the three most frequently used stations are Station 4 (S4), Station 9 (S9), and Station 15 (S15). At S4, it is observed that on weekdays there is a peak of traffic in the morning and afternoon, while on the weekends the traffic decreases with no peak hours. At S9, the peak hours are early in the morning and late in the afternoon, whereas on the weekends the traffic is gathered during midday hours where there is a peak of traffic. The most used metro station seems to be S15 throughout the week. The passengers flow follows a certain pattern on weekdays. There are morning peak hours except for Fridays where most of the traffic is in the afternoon. On Saturdays, S15 is mostly used around 16.00.

**Figure 3:**
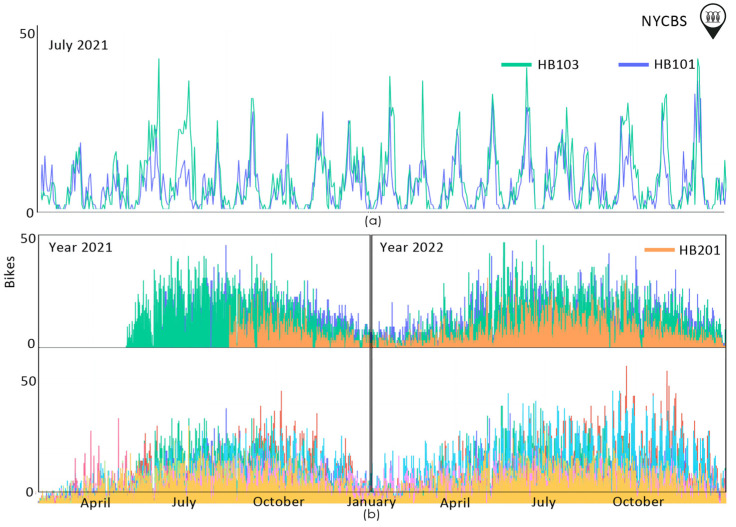
The transportation flow through time in NYCBS system dataset (**a**) for July 2021; (**b**) for years 2021 and 2022. There is a yearly pattern. During the winter months, there is a decline in bike demand, which starts to rise again from May onwards. The peak demand is observed during the summer months. Different color lines refer to different stations.

The transportation flow through time in NYCBS is presented in Figure 3. As we can see, there is a yearly pattern. Ιn the winter months, bike demand falls and starts to rise again from May onwards. Peak demand is found in the summer months. For instance, in July 2021, the peak hours for the most frequently used stations (HB101, HB103), are from 17.00 to 20.00 in the afternoon. There are also stations that are not in use throughout the year. For example, stations HB101, 102, 103, have started their usage in May 2021, whereas HB103 interrupts its operation at certain intervals.

The operation management of the stations, their interruption for certain periods of time or permanently when demand is low, or the creation of new stations due to high traffic in the area, are a matter of critical importance for the organization and the efficient operation of the system. Therefore, knowing the traffic flow of people in a mode of transportation like the metro and of bicycles in a bike rental system, is essential to all these issues in order that they may be organized and run as efficiently as possible.

**Figure 4:**
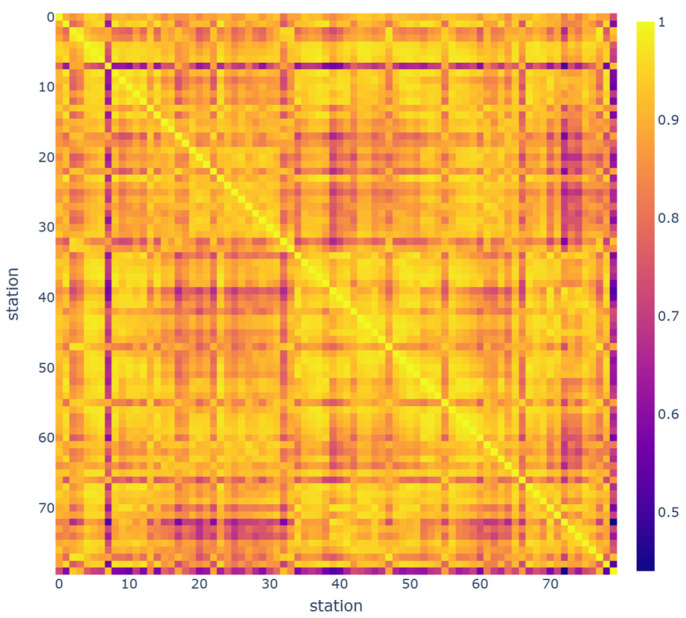
Heatmap that visualizes the spatial dependency between stations, in Hangzhou metro system dataset. The majority of Hangzhou metro stations exhibit a high level of dependency on each other, with correlation values closer to 1.

**Figure 5:**
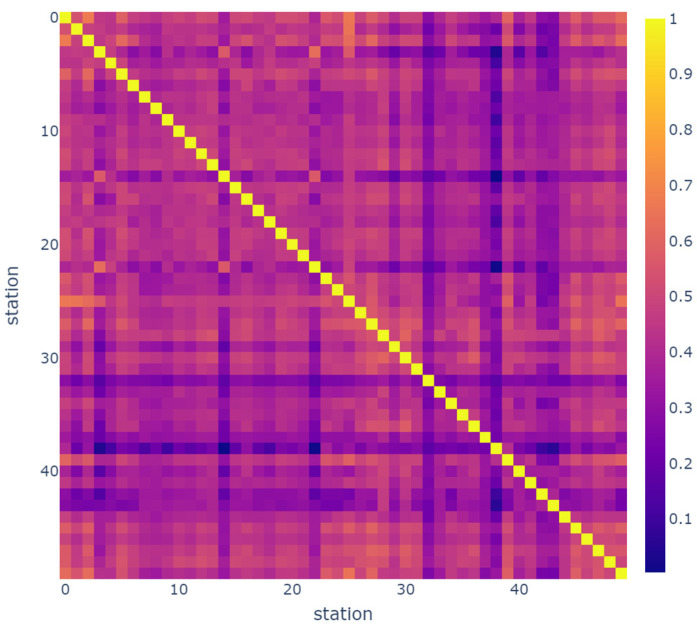
Heatmap that visualizes the spatial dependency between stations, in NYCBS system dataset. The low correlation values, which are close to zero, can be attributed to the unstructured nature of the bike stations network.

In order to see the spatial dependency between stations, the correlation matrix is calculated and is visualized by a heatmap presented in Figure 4 and Figure 5 for Hangzhou metro and NYCBS datasets, respectively. The color inside each cell indicates the correlation coefficient of the relationship. As the color indicates, most of the Hangzhou metro stations are dependant on each other to a great extent, in contrast with NYCBS which are less correlated. This is reasonable, since the metro network is more structured and the transportation flow has more specific patterns.

### 4.3. Data Preparation

Hangzhou Metro System: The dataset has been processed in order to have the incoming and outcoming passenger number per time and station. The time window has been set to 15 min. As there are differing scales in values, the input data are normalized, based on mean value and standard deviation.

NY City Bike System (NYCBS): The initial data are transformed in order to have the number of bikes per time and station, with 60 min interval. By using the start and end station of each route, we create the spatial connections between stations. We are not based on their physical connection but on the connection based on the usage of bikes and the trips between stations, which is more realistic and practically useful. The data are normalized, based on mean value and standard deviation.

Both datasets are split into two parts to create a training set and a testing set. The model is trained using 80% of the rows, while the remaining 20% is assigned to the test set.

### 4.4. Baselines

In this subsection, the performance of our proposed model is evaluated. After extensive experiments on machine and deep learning methods in order to manage the inclusion of pertinent models in our work and achieve their optimal performance, we finally chose four methods as a benchmark to our proposed model. The experiments are carried out on Hangzhou metro and NY city bike system. The chosen models are the following:

- Multi-Output Random Forest Regression (MO-RF) [42]: MO-RF is a multi-output regression method which utilizes MultiOutputRegressor. This approach involves fitting a single regressor for each target variable. Since each target variable is represented by its own regressor, it is possible to obtain insights about the target by examining its corresponding regressor. However, MultiOutputRegressor cannot benefit from correlations between targets since it fits only one regressor per target [43].
- Long Short-Term Memory (LSTM) [39]: LSTM neural network is widely used in time series forecasting tasks due to its strong capacity to discover and utilize the information concealed in time series sequences.
- Gated Recurrent Unit (GRU) [44]: GRU network generally performs similarly to LSTM on many tasks, but in some cases, GRU seems to outperform LSTM, as it is faster to train, has a simpler structure and fewer parameters than LSTM, and performs better on large datasets or sequences.
- Transformers [45]: Transformers is a technique that stands out for its use of self-attention and differential weighting of the importance of each component of the input data. Transformers process the entire input all at once.

In addition to the aforementioned models, we incorporate several existing advanced methods proposed in other literature to evaluate the predictive capabilities of our proposed model. The inclusion of these alternative methods in our evaluation enables us to conduct a comprehensive assessment of the performance and effectiveness of our approach.

The models used as benchmarks for passenger flow estimation in Hangzhou metro, are the following:

- Spatial-Temporal Hypergraph Graph Convolutional Network (STHGCN) / Dynamic Spatial-Temporal Hypergraph Graph Convolutional Network (DSTHGCN) [35]: This work proposes a dynamic spatial-temporal hypergraph neural network to forecast passenger flow.
- Split-Attention Relational Graph Convolutional Network (SARGCN) [36]: This approach combines the Relational Graph Convolutional Network (R-GCN), split-attention mechanism, and LSTM to analyze the spatial-temporal correlations and dependencies between passenger inflow and outflow.
- Parallel Bidirectional Gated Recurrent Unit (PB-GRU) [46]: A deep learning model composed of parallel multi-graph convolution and stacked bidirectional unidirectional gated recurrent unit for metro passenger volume prediction.
- Multi-View Multi-Attention Graph Neural Network (AMGC-AT) [37]: A multi-view convolution module with a spatial-temporal self-attention module and a gated convolution network for traffic flow forecasting.
- Spatial-Temporal Dynamic Graph Relational Learning model (STDGRL) [47]: A spatial-temporal dynamic graph relational learning model for urban metro flow prediction.

The models used as benchmarks for bike demand estimation in NY Citi Bike system are the following:

- Spatial-Temporal Graph Neural Network (ST-GNN) [32]: A deep learning technique is employed in a two-phase framework to accurately predict city-wide bike demands and effectively rebalance bike stations.
- Attention-based Spatial-Temporal Graph Convolutional Network (AST-GCN) [33]: In order to predict the number of available bikes in bike-sharing systems in cities, an attention-based mechanism is introduced to enhance the performance of an ST-GCN.
- Graph Convolutional Network Multigraph (GCN-Multigraph) [48]: GCN-Multigraph is a model which predicts the bike flow at the station level by analyzing the bike-sharing system from a graph perspective.
- Multi-View Graph Convolutional Network (MVGCN) [49]: A multi-view graph convolutional network for forecasting crowd flow.

### 4.5. Evaluation Metrics

To evaluate the performance of our method in comparison with the baseline models, three evaluation metrics are used: Average Absolute Error (MAE), Root Mean Square Error (RMSE), and Mean Absolute Percentage Error (MAPE). In the context of machine and deep learning:

*MAE* refers to the magnitude of difference between the prediction of an observation and the true value of that observation.

(10) $$MAE=\frac{1}{N}\overset{N}{\underset{i=1}{\sum }}\left|f\left(i\right)-h\left(i\right)\right|$$

*RMSE* shows how far estimations fall from measured true values using Euclidean distance.

(11) $$RMSE=\sqrt{\frac{1}{N}\;\sum_{i=1}^{N}{\left(f\left(i\right)-h\left(i\right)\right)}^{2}}$$

*MAPE* refers to the average absolute percent error for each time period minus actual values divided by actual values.

(12) $$MAPE=\frac{1}{N}\sum_{1}^{n}\left|\frac{f\left(i\right)-h\left(i\right)}{h\left(i\right)}\;\right|\;$$

where *f*(*i*) is the predicted value and *h*(*i*) is the actual value at the time *i*. Moreover, *N* is the total number of the features in dataset.

### 4.6. Model Hyper-Parameters

Our model utilizes three previous time stamps of historical data and predicts the transportation flow at the next one time stamp.

The experiments are carried out on a system with an Intel Core i7 CPU @ 3.4 GHz 3.40 GHz processor and 8 GB RAM. The model is developed based on Python 3.9.7, Tensorflow, and Keras 2.8.0.

The selection range of hyper-parameter values for both datasets is determined through extensive experiments in order to produce the optimal results for the evaluation metrics.

**Figure 6:**
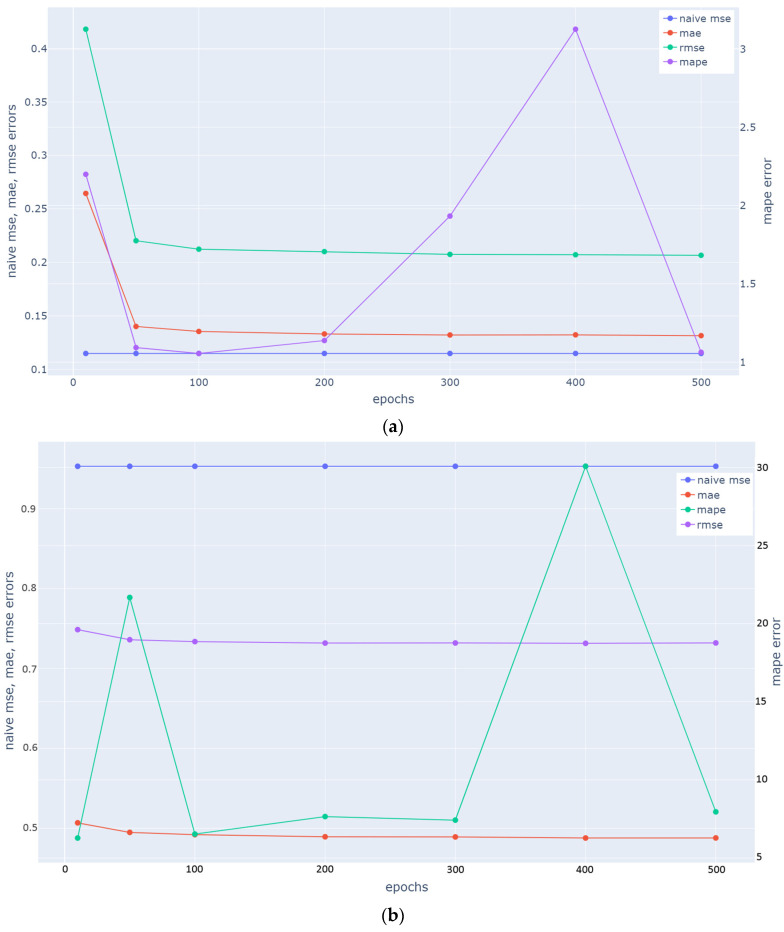
Mean Absolute Error (MAE), Root Mean Square Error (RMSE), and Mean Absolute Percentage Error (MAPE) in accordance with epoch values used in ST-GCRN for (**a**) Hangzhou metro system dataset; (**b**) NYCBS dataset. As the number of epochs increases, we can observe significant fluctuations in the MAPE, while the MAE and RMSE do not appear to be affected.

**Figure 7:**
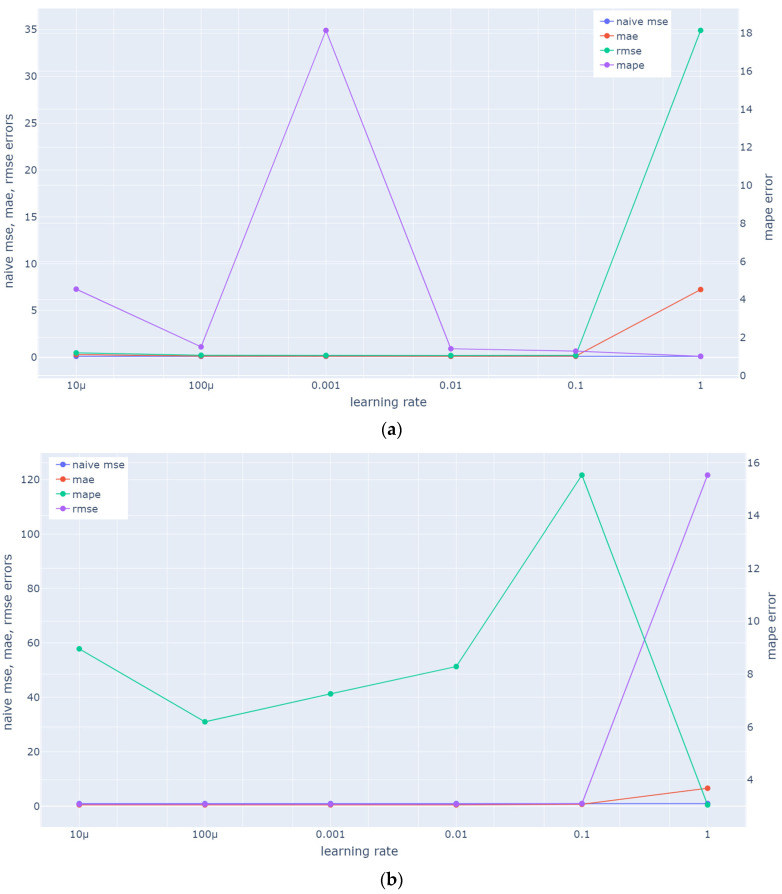
Mean Absolute Error (MAE), Root Mean Square Error (RMSE), and Mean Absolute Percentage Error (MAPE) in accordance with learning rate values used in ST-GCRN for (**a**) Hangzhou metro system dataset; (**b**) NYCBS dataset. With an increase in the learning rate, the MAPE demonstrates relatively less variation compared to an increase in epochs. However, it still exhibits a sharp increase followed by a decrease when the rate reaches a high value in Hangzhou metro system (**a**).

Hangzhou Metro System: To optimize all metrics and achieve a balanced result, we select a value of 100 for the epochs and a batch size value of 64. As depicted in Figure 6a, this decision is based on the lowest MAPE value achieved, considering that additional epochs above 100 do not yield any significant improvement in the other metrics (MAE, MSE). Similarly, as depicted in Figure 7a, the learning rate value that is selected is 10^−4^ in order to ensure the minimization of all metrics. Root mean squared propagation [50] is used as optimizer and LSTM units are set to 256.

NY City Bike System (NYCBS): To optimize all metrics and achieve a balanced result, we select a value of 200 for the epochs and a batch size value of 64. As depicted in Figure 6b, this decision is based on the lowest RMSE and MAE although MAPE value has a small increase. Similarly, as depicted in Figure 7b, the learning rate value that is selected is 10^−4^, the value at which all metrics reach their minimum. According to [51], Adam method is “computationally efficient, has little memory requirement, invariant to diagonal rescaling of gradients, and is well suited for problems that are large in terms of data/parameters”. LSTM units are set to 128.

The hyper-parameters of the model are summarized in Table 6 for both datasets.

## 5. Experimental Results

### 5.1. Hyper-Parameter Tuning

In Figure 6 and Figure 7, we can observe how two basic parameters (number of epochs and learning rate) of the model for Hangzhou metro system and NYCBS dataset, influence the metrics’ values (MAE, RMSE, MAPE).

We can observe that MAPE fluctuates significantly as the number of epochs increases, while MAE and RMSE do not seem to be affected. The same is the case with the learning rate, where, as learning rate increases, the MAPE has a smaller variation compared to the increase in epochs, but still shows a sharp increase and then a decrease when the rate reaches a high value, in Hangzhou metro system. The loss increases as the learning rate is increased since the loss “bounces around” and even diverges from the minima due to the parameter adjustments. A large learning rate typically accelerates the learning process but results in an inefficient final set of weights. A model may learn a more ideal set of weights with a smaller learning rate, but training may take significantly longer. Τherefore, as expected in our case, a learning rate that is quite large, leads to an unstable MAPE value as the average loss increases [52].

### 5.2. Flow Estimation Results

**Figure 8:**
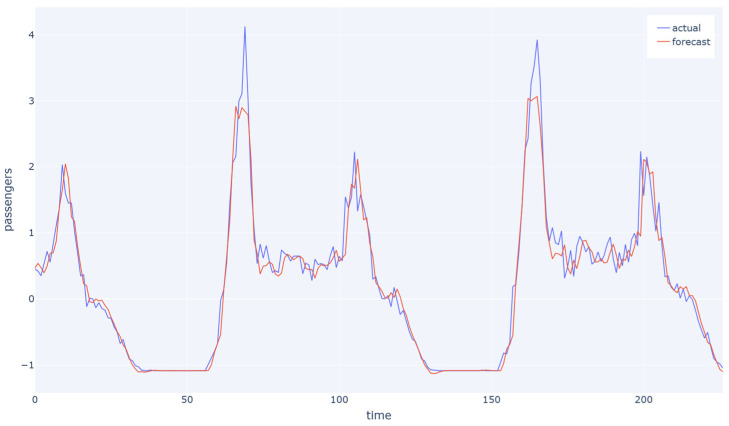
The average values of the actual transportation flow for one station over time in accordance with the predicted values for Hangzhou metro system dataset with a sample rate of 15 min. The model is able to capture the overall flow trend.

**Figure 9:**
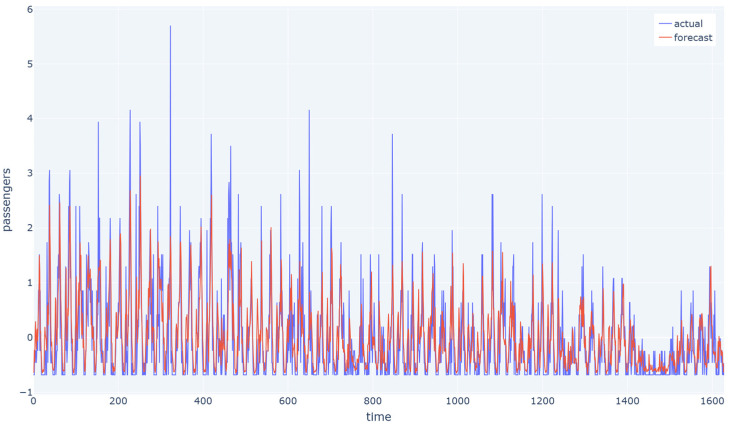
The average values of the actual transportation flow for one station over time in accordance with the predicted values for NYCBS dataset with a sample rate of 60 min. Due to the unstructured nature of the dataset, the model is able to capture the overall flow trend, but it struggled to accurately predict certain peak values.

In Figure 8 and Figure 9, the blue line represents the average values of the actual transportation flow for one station over time, while the red line represents the predicted values. In the Hangzhou metro dataset, it is clear that the model was able to capture the overall flow trend more accurately than in the NYCBS dataset and this is reasonable and expected since the latter is a more unstructured network with more unpredictable traffic flow.

**Figure 10:**
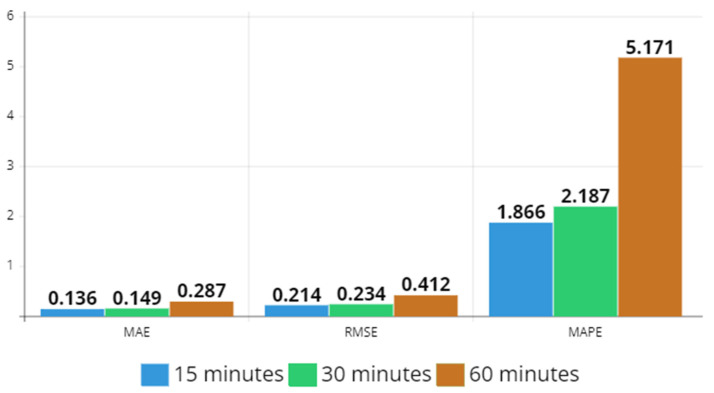
MAE, RMSE, and MAPE values for 15 min, 30 min, and 60 min time intervals for Hangzhou metro system dataset. Each error value increases as the sampling rate increases.

**Figure 11:**
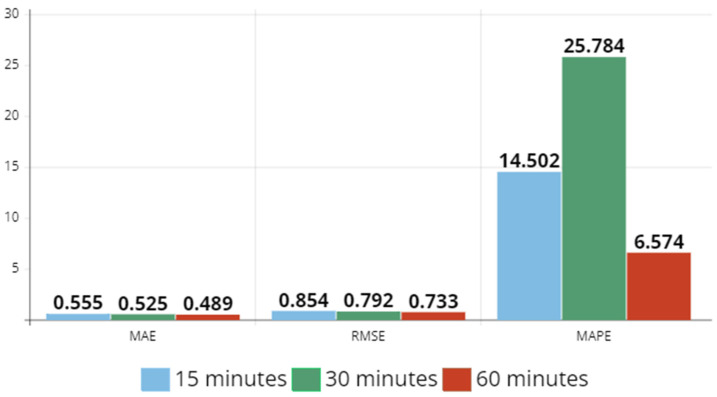
MAE, RMSE, and MAPE values for 15 min, 30 min, and 60 min time intervals for NYCBS dataset. Each error value of MAE and RMSE decrease as the sampling rate increases.

In Figure 10 and Figure 11, MAE, RMSE, and MAPE values are depicted for 15 min, 30 min, and 60 min time intervals, for both datasets. In Hangzhou metro dataset, each error value increases as the sampling rate increases, whereas in NYCBS dataset, MAE and RMSE decrease. Therefore, in a dynamic system like the bike system where the relationships between stations are not predetermined and change dynamically, the sampling rate must be higher than in a more static system like the subway in order to make a better estimation in transportation flow.

### 5.3. Comparison with Alternative Models

We compare ST-GCRN to all baselines with 15 min, 30 min, and 1 h prediction time intervals, for short-term, middle-term, and long-term estimation, respectively. In these experiments, our model utilizes three previous time stamps of historical data and predicts the transportation flow at the next one time stamp. Table 7 and Table 8 show the comparison of ST-GCRN with existing models from previous works on Hangzhou metro and NYCBS datasets, respectively. The bold data indicate the best results. As mentioned in Section 5.1, MAPE is prone to variations; therefore, we prefer to focus on MAE and RMSE error values minimization.

The main inferences from the estimation findings are distilled as follows:

1. ST-GCRN can achieve the lowest MAE and RMSE, among all the existing proposed frameworks, as presented in Table 7 and Table 8 with an error decrease of 98%, in both datasets.
2. As MAPE is concerned, our framework outperforms the other frameworks except for STDGRL in Hangzhou metro dataset with a small difference though.

Table 9 and Table 10 present a comparison between ST-GCRN and various machine learning and deep learning models created specifically for this study, using the Hangzhou metro and NYCBS datasets. In Table 9, the symbol “*” indicates that the prices are excessively high and surpass the compared prices of the other models.

1. It is observed that RF performed marginally better than our proposed framework only in Hangzhou metro dataset due to its relative small amount of data as well as the structured spatial layout of the station network. In all other cases, it is clear that ST-GCRN outperforms the other models in terms of MAE and RMSE values.
2. LSTM and GRU, in both datasets, have relative performance as MAE and RMSE indicate and only in MAPE they have a substantial difference.
3. Transformers do not perform well compared with the other models. Although the research community has tried to develop Transformer variants for longer sequences and these methods are quite efficient, their preconceived notions about the structure of the attention matrix, acquired through training, may not always be suitable for tasks beyond Natural Language Processing (NLP) [53]. A very high MAPE value is observed that seems to be due to zero values indicating no connections between stations as well as due to big variance in data values as depicted in Figure 2 and Figure 3.

### 5.4. Modeling Results with Different Estimation Horizons

In this subsection, we describe the results that arise from various experiments on different estimation horizons. Table 11 shows the change in MAE, RMSE, and MAPE values at different estimation horizons, i.e., one, two, or three time steps ahead from three previous samples.

1. It can be seen that ST-GCRN obtains the best prediction performance under all evaluation metrics for all estimation horizons, proving the effectiveness of ST-GCRN in spatial-temporal traffic forecasting.
2. With little variation in the error value, we are able to predict also the second and the third next time steps from the previous three samples.
3. The best performance was accomplished by predicting two next time steps and one next time step in Hangzhou metro and NYCBS, respectively. Once again, the structured form of the metro network allows for a longer term forecasting.

### 5.5. Comparison between the Two Datasets

**Figure 12:**
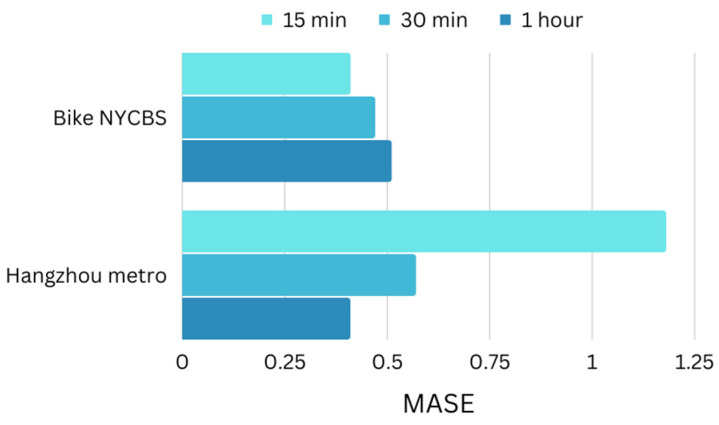
Performance of the two datasets compared with each other with 15 min, 30 min, and 1 h prediction time intervals based on Mean Absolute Scaled Error (MASE). The decrease in the sampling rate of the Hangzhou metro system leads to a reduction in the MASE, whereas in the NYCB system results in a decrease in the MASE value.

**Figure 13:**
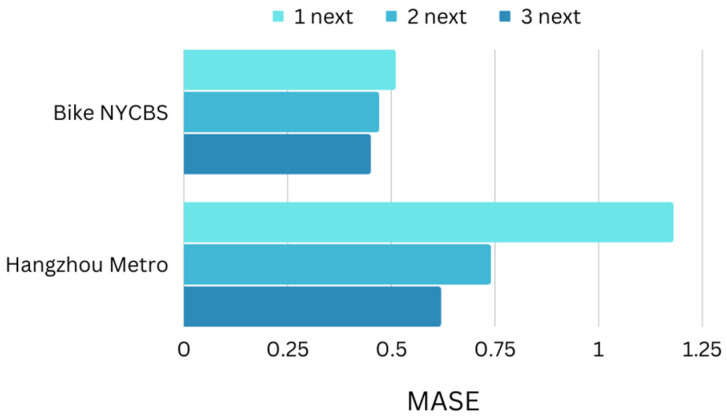
Performance of the two datasets compared with each other with estimation horizons of one, two, and three time steps ahead based on Mean Absolute Scaled Error (MASE). NYCBS dataset performs better in all three cases of one, two, and three time steps ahead from three previous samples.

The performance of the model is assessed using two datasets with distinct characteristics, as described in Section 4.1 Mean Absolute Scaled Error (MASE) is a metric used in time series estimation to evaluate the accuracy of forecasts produced by an algorithm. Its value is given by the ratio of MAE for algorithm and MAE of naïve forecast and it provides an insight into how well a forecasting algorithm is performing in comparison to this naïve forecast. If the value of MASE is greater than one (1), it suggests that the algorithm is performing poorly. By applying ST-GCRN model to the datasets we use for the model’s evaluation, as presented in Figure 12 and Figure 13, the following points are observed:

1. The decrease in the sampling rate of the Hangzhou metro system leads to a reduction in the MASE. On the other hand, it has been noticed that in the NYCB system, where the connections between stations are dynamically established, an increase in the sampling rate results in a decrease in the MASE value.
2. As far as long-term horizon transportation flow estimation is concerned, NYCB dataset performs better in all three cases of one, two, and three time steps ahead from three previous samples.

### 5.6. Computation Efficiency

The experiments are carried out on a system with an Intel Core i7 CPU @ 3.4 GHz 3.40 GHz processor and 8 GB RAM. The training time needs about 20.59 s/epoch for Hangzhou metro and 242.45 for NYCBS, while the inference time only takes a few seconds as depicted in Table 12. Given that the training process is conducted offline, the current running efficiency is considered sufficient for real-life metro and bike flow prediction systems.

## 6. Conclusions

In conclusion, this paper introduces a Spatial-Temporal Graph Convolutional Recurrent Network (ST-GCRN), as a solution to accurately estimate transportation flow. The technique effectively detects dynamic spatial correlations between stations and performs long-term estimation. By utilizing a graph convolutional network and LSTM neural network, the model captures both spatial and temporal dependencies in transportation flow data. A dynamic relation graph is proposed to capture the dynamic spatial dependencies between stations.

The effectiveness of the proposed framework is evaluated on real-world datasets from Hangzhou metro and New York City’s Citi Bike system. The experiments demonstrated that ST-GCRN outperform current state-of-the-art baselines, reducing estimation errors by 98% in the metro system and 63% in the bike-sharing system. The model not only estimates future transportation flow in short and long terms, but also in various time horizons, improving accuracy and practicality compared to existing baselines.

The evaluation results show superior prediction performance of ST-GCRN across all estimation horizons, surpassing other models based on various evaluation metrics. This highlights the effectiveness of ST-GCRN in spatial-temporal traffic forecasting.

Despite the numerous advantages offered by the Spatial-Temporal Graph Convolutional Recurrent Network (ST-GCRN) model, such as its ability to capture temporal dependencies, incorporate relationships and dependencies among entities in graph-based data, handle non-linear relationships, and enable scalability and efficiency for large-scale datasets through parallel processing, it is crucial to acknowledge its limitations. Firstly, implementing and training the ST-GCRN model can be complex, requiring substantial computational resources and a significant amount of data to effectively learn and generalize patterns. Additionally, interpreting the underlying relationships and providing meaningful explanations for the model’s predictions can be challenging. 

To effectively tackle these concerns in future studies, our plan is to investigate the integration of external contextual factors, such as climate conditions, economic factors, and social events. This inclusion of additional variables is anticipated to improve the model’s ability to make accurate estimations of transportation flow by considering the impact of various external factors on the system. As a result, we expect to enhance the overall generalizability of the proposed model.

Furthermore, our intention is to incorporate the prediction of congestion patterns in transportation zones or busy areas. This involves forecasting or estimating the levels and patterns of traffic congestion in specific stations or transportation media that frequently experience high traffic volume or congestion. The objective of this prediction is to anticipate when and how severe congestion will occur in these areas, providing valuable insights into the dynamics of transportation flow. As a result, we can facilitate proactive measures to manage and alleviate congestion. To achieve this, we will analyze historical transportation data, including information on transportation volume, speed, and density, as well as other relevant factors, such as road infrastructure, events, and weather conditions. By leveraging these data, we will develop predictive models capable of forecasting congestion patterns. These models will enable transportation planners, traffic management authorities, and commuters to make well-informed decisions regarding route planning, transportation management strategies, and travel time estimation.

Finally, our future plans involve integrating explainable machine learning techniques to enhance the interpretability of our models and provide meaningful explanations for their predictions. This could involve incorporating methods, such as feature importance analysis, rule-based models, surrogate models, or attention mechanisms to make our models more interpretable. By incorporating explainable machine learning into our upcoming research, our objective is to improve transparency, trust, and accountability in our models. We believe that this approach will not only enhance the comprehension of our predictions, but also offer valuable insights for stakeholders and users, empowering them to make informed decisions based on the model’s output.

## Figures and Tables

**Table 1:** The topological structure of the stations network in Hangzhou metro and NYCBS.

	Hangzhou Metro	Bike NYCBS
Number of nodes	80	50
Number of edges	6320	2450

**Table 2:** Description of datasets used in the evaluation of ST-GCRN.

Dataset	Hangzhou Metro	NYCBS
City	Hangzhou	New York
Number of stations	81	50
Number of samples	2293	14,848
Time Interval	15 min	60 min

**Table 3:** Description of dataset-specific features that each system has in real world.

Hangzhou Metro	NYCBS
More structured stations network	More unstructured stations network
More difficult to reformat stations position	More flexible to change stations position
Mostly predetermined traffic patterns	More random station usage
Physical connections between stations	Dynamic connections between stations
Smaller time intervals in metro usage	Bigger time intervals in bike usage
Smaller number of stations	Greater number of stations

**Table 4:** Description of datasets features (columns) in Hangzhou metro.

Hangzhou Metro	
time	Time stamp
Station ID	ID of the station
status	0: outcoming passengers flow
	1: incoming passengers flow
userID	ID of the user

**Table 5:** Description of datasets features (columns) in NYCBS.

NYCBS	
time	Time stamp
bikeID	ID of the bike
StartStationID	The start station of the trip ID
EndStationID	The end station of the trip ID

**Table 6:** The hyper-parameters of the model that were finally selected after extensive experiments, for both datasets.

Parameters	Hangzhou Metro	Bike NYCBS
Batch size	64	64
Epochs	100	200
Optimizer	RMSProp	Adam
Learning rate	10^−4^	10^−4^
LSTM units	256	128

**Table 7:** Comparison of ST-GCRN with existing models from previous works proposed in other literature, on Hangzhou metro dataset.

Models	15 min			30 min			60 min		
	MAE	RMSE	MAPE	MAE	RMSE	MAPE	MAE	RMSE	MAPE
STHGCN/DSTHGCN [32]	11.400/11.080	26.770/26.990	-	12.900/13.010	28.220/27.840	-	-	-	-
SARGCN [33]	22.480	36.220	13.940	23.460	37.830	14.990	25.290	41.590	17.600
PB-GRU [43]	22.130	36.550	13.300	22.900	38.330	13.750	23.910	40.020	14.870
AMGC-AT [34]	19.670	31.240	-	30.680	50.790	-	-	-	-
STDGRL [44]	23.720	46.860	0.210	24.370	49.290	0.210	26.580	57.390	0.230
ST-GCRN (proposed)	0.136	0.214	1.866	0.149	0.234	2.187	0.287	0.412	5.171

**Table 8:** Comparison of ST-GCRN with existing models from previous works proposed in other literature, on NYCBS dataset.

Models	15 min			30 min			60 min		
	MAE	RMSE	MAPE	MAE	RMSE	MAPE	MAE	RMSE	MAPE
ST-GNN [29]	-	-	-	-	-	-	2.580	3.860	7.900
AST-GCN [30]	-	-	-	1.880	-	-	-	-	-
GCN-Multigraph [45]	-	-	-	-	-	-	-	4.003	-
MVGCN [46]	-	-	-	-	-	-	2.600	4.150	-
ST-GCRN (proposed)	0.555	0.854	14.502	0.525	0.792	25.784	0.489	0.733	6.574

**Table 9:** Performance comparison between ST-GCRN and various machine learning and deep learning models on Hangzhou metro system dataset.

Models	15 min			30 min			60 min		
	MAE	RMSE	MAPE	MAE	RMSE	MAPE	MAE	RMSE	MAPE
MO-RF [39]	0.117	0.176	1.451	0.111	0.179	1.200	0.172	0.286	0.929
LSTM [36]	0.174	0.272	1.362	0.197	0.295	2.783	0.490	0.700	4.821
GRU [41]	0.172	0.266	1.895	0.189	0.293	6.218	0.397	0.579	2.709
Transformers [42]	0.766	1.028	*	0.760	1.022	*	0.767	1.019	*
ST-GCRN (proposed)	0.136	0.214	1.866	0.149	0.234	2.187	0.287	0.412	5.171

* The prices are excessively high and surpass the compared prices of the other models.

**Table 10:** Performance comparison between ST-GCRN and various machine learning and deep learning models on NYCBS dataset.

Models	15 min			30 min			60 min		
	MAE	RMSE	MAPE	MAE	RMSE	MAPE	MAE	RMSE	MAPE
MO-RF [39]	0.574	0.866	43.726	0.542	0.797	10.623	0.508	0.738	6.655
LSTM [36]	0.632	0.880	3.876	0.575	0.824	12.783	0.566	0.798	6.185
GRU [41]	0.663	0.890	5.127	0.596	0.834	6.693	0.560	0.795	6.122
Transformers [42]	0.700	0.923	79.907	0.692	0.920	76.385	0.693	0.920	103.553
ST-GCRN (proposed)	0.555	0.854	14.502	0.525	0.792	25.784	0.489	0.733	6.574

**Table 11:** MAE, RMSE, and MAPE values on different estimation horizons.

	15 min			60 min		
Different Estimation Horizons	Hangzhou Metro			Bike NYCBS		
	MAE	RMSE	MAPE	MAE	RMSE	MAPE
one next from three previous samples	0.555	0.854	14.502	0.525	0.792	25.784
two next from three previous samples	0.215	0.356	2.484	0.543	0.809	7.686
three next from three previous samples	0.335	0.553	2.962	0.606	0.893	9.039

**Table 12:** Number of parameters, inference and training time for Hangzhou metro and NYCBS.

	Hangzhou Metro	Bike NYCBS
Number of parameters	283,915	76,427
Inference time (seconds)	1.14	9.97
Training time (seconds/epoch)	20.59	242.45

## Data Availability

Not applicable.
